# Common and Potential Emerging Foodborne Viruses: A Comprehensive Review

**DOI:** 10.3390/life14020190

**Published:** 2024-01-28

**Authors:** Amin N. Olaimat, Asma’ O. Taybeh, Anas Al-Nabulsi, Murad Al-Holy, Ma’mon M. Hatmal, Jihad Alzyoud, Iman Aolymat, Mahmoud H. Abughoush, Hafiz Shahbaz, Anas Alzyoud, Tareq Osaili, Mutamed Ayyash, Kevin M. Coombs, Richard Holley

**Affiliations:** 1 Department of Clinical Nutrition and Dietetics, Faculty of Applied Medical Sciences, The Hashemite University, P.O. Box 330127, Zarqa 13133, Jordan; murad@hu.edu.jo (M.A.-H.); mahmoud.abughoush@aau.ac.ae (M.H.A.); 2 Department of Nutrition and Food Technology, Faculty of Agriculture, Jordan University of Science and Technology, P.O. Box 3030, Irbid 22110, Jordan; aotaybeh19@agr.just.edu.jo (A.O.T.); anas_nabulsi@just.edu.jo (A.A.-N.); tosaili@sharjah.ac.ae (T.O.); 3 Department of Medical Laboratory Sciences, Faculty of Applied Medical Sciences, The Hashemite University, P.O. Box 330127, Zarqa 13133, Jordan; mamon@hu.edu.jo; 4 Department of Anatomy, Physiology and Biochemistry, Faculty of Medicine, The Hashemite University, P.O. Box 330127, Zarqa 13133, Jordan; jihada@hu.edu.jo (J.A.); imank@hu.edu.jo (I.A.); 5 Science of Nutrition and Dietetics Program, College of Pharmacy, Al Ain University, Abu Dhabi P.O. Box 64141, United Arab Emirates; 6 Department of Food Science and Human Nutrition, University of Veterinary and Animal Sciences, Lahore 54000, Pakistan; shahbaz.uaf@gmail.com; 7 Faculty of Medicine, The Hashemite University, P.O. Box 330127, Zarqa 13133, Jordan; anasabedalbaset12321@gmail.com; 8 Department of Clinical Nutrition and Dietetics, College of Health Sciences, University of Sharjah, Sharjah P.O. Box 27272, United Arab Emirates; 9 Department of Food Science, College of Agriculture and Veterinary Medicine, United Arab Emirates University, P.O. Box 15551, Al Ain 53000, United Arab Emirates; mutamed.ayyash@uaeu.ac.ae; 10 Department of Medical Microbiology and Infectious Diseases, Max Rady College of Medicine, University of Manitoba, Winnipeg, MB R3E 0J9, Canada; kevin.coombs@umanitoba.ca; 11 Department of Food and Human Nutritional Sciences, University of Manitoba, Winnipeg, MB R3T 2N2, Canada; rick.holley@umanitoba.ca

**Keywords:** norovirus, rotavirus, hepatitis, adenovirus, astrovirus, acute viral gastroenteritis, enteric viruses, foodborne infections, food safety

## Abstract

Human viruses and viruses from animals can cause illnesses in humans after the consumption of contaminated food or water. Contamination may occur during preparation by infected food handlers, during food production because of unsuitably controlled working conditions, or following the consumption of animal-based foods contaminated by a zoonotic virus. This review discussed the recent information available on the general and clinical characteristics of viruses, viral foodborne outbreaks and control strategies to prevent the viral contamination of food products and water. Viruses are responsible for the greatest number of illnesses from outbreaks caused by food, and risk assessment experts regard them as a high food safety priority. This concern is well founded, since a significant increase in viral foodborne outbreaks has occurred over the past 20 years. Norovirus, hepatitis A and E viruses, rotavirus, astrovirus, adenovirus, and sapovirus are the major common viruses associated with water or foodborne illness outbreaks. It is also suspected that many human viruses including Aichi virus, Nipah virus, tick-borne encephalitis virus, H5N1 avian influenza viruses, and coronaviruses (SARS-CoV-1, SARS-CoV-2 and MERS-CoV) also have the potential to be transmitted via food products. It is evident that the adoption of strict hygienic food processing measures from farm to table is required to prevent viruses from contaminating our food.

## 1. Introduction

Viruses cannot grow on lifeless substrates since they are obligate intracellular parasites that require living cells in which to replicate. At the same time, all currently known viruses are host-specific. Worldwide, it is becoming apparent that foodborne illnesses are increasingly contributing to human morbidity in spite of the fact that approaches to reverse this trend are available. More than 200 diseases in humans can occur following exposure to food contaminated by bacteria, viruses, parasites or chemicals. Each year, approximately one in ten people get infected after eating contaminated food worldwide, and this represents 600 million foodborne illnesses, and ultimately 420,000 deaths [1]. Although the United States (US) has one of the safest food supplies in the world, the federal government estimates that more than 48 million foodborne cases with 128,000 hospitalizations and 3000 deaths occur each year. This means that one in six Americans suffer at least one episode of foodborne illness each year [2,3].

Enteric viruses are represented by those genera that invade and replicate in the mucosa or epithelial cell lining of the small intestine [4]. Although viruses cannot grow in food, they are associated with large numbers of foodborne outbreaks and caused 59 % of all foodborne illnesses which occurred in the US according to CDC [2,5]. Noroviruses were the leading cause of foodborne illnesses, with about 5.46 million cases annually, and they are considered the second and fourth leading cause of hospitalizations and deaths, respectively [2,5,6]. Five enteric viruses, namely norovirus, rotavirus, hepatitis A virus, astrovirus, and sapovirus, were among the 31 major foodborne pathogens identified by the CDC [2]. Other viruses, such as adenovirus and hepatitis E virus, are also associated with foodborne diseases [7]. Zoonotic viruses that are harbored by animals and birds including tick-borne encephalitis virus, coronaviruses, ebola virus, avian influenza virus, nipah virus, and aichi virus have the potential to be transmitted via foods and cause foodborne illnesses [8]. 

The contamination of food with viruses may be managed either by inactivation or by preventing viral occurrence [9,10]. Effective antiviral measures involve: implementing specific controls for raw materials as well as for food production; adopting appropriate food safety management systems such as Good Agricultural Practices (GAP) and Good Manufacturing Practices (GMP) from farm to fork; food-handling education; effective sanitation measures; and adequate hand hygiene along with suitable strategies to manage ill workers [11]. In addition, recent preservation technologies including irradiation, pulsed electric field, high pressure processing, ultra violet (UV) light, and cold plasma can be used to inactivate viruses in foods [12,13,14,15,16]. The objective of this review is to discuss the information available on the general and clinical characteristics of viruses, viral foodborne outbreaks, and control strategies to prevent the viral contamination of food products and water.

## 2. Common Foodborne Viruses

Foodborne viruses are increasingly recognized as causes of illnesses in humans. Enteric viruses persist well in the environment, on different surfaces and preparation areas in food service establishments and allied industries, as well as on human hands [17]. 

### 2.1. Norovirus

Noroviruses, a non-enveloped, positive-sense, single-stranded (ss)RNA viral group, belongs to the family *Caliciviridae*. Previously, noroviruses were named Norwalk or Norwalk-like viruses after the original Norwalk strain caused an outbreak of gastroenteritis in a school in 1968 in Norwalk, Ohio [18]. Currently, noroviruses are divided into 10 genogroups (GI to GX). GI, GII, GIV, GVIII and GIX are identified to infect humans [19,20]. GII.4 is more often spread via person-to-person contact. In comparison, non-GII.4 genotypes, such as GI.3, GI.6, GI.7, GII.3, GII.6 and GII.12, are more often transmitted to humans via foodborne routes [21]. Norovirus genotypes GII.2 and GII.4 are mostly implicated in outbreaks of gastroenteritis, especially in closed institutions such as schools, nursing homes and summer camps [22]. However, genogroup GI strains are more often linked to waterborne outbreaks of norovirus [23]. In the United States, 24,995 single-state norovirus outbreaks were reported between 2009 and 2019 [24]. In another cross-continental study involving 16 countries, the predominance of GII.4 (˃50% of cases), especially among adults, was shown, whereas genotypes GII.2, GII.3 and GII.6 were more common among children [25]. In China, it was reported that noroviruses are the predominant cause of gastroenteritis among young children < 2 y and among those aged ≥ 65 y [26].

The human norovirus infection is self-limiting; however, worldwide, it is usually associated with mortality among the immunocompromised patients, the elderly, and children [27,28]. Norovirus shows considerable variability in the risk of infection and the severity of the symptoms depending on its genotype, host susceptibility, and the dose of the ingested virus [29]. Noroviruses cause highly contagious disease, which is believed to represent almost 20% of all acute gastroenteritis cases, with about 20,000 deaths globally [30,31,32]. The general and clinical characteristics of noroviruses are listed in Table 1.

Norovirus spread can take place via direct contact with an infected individual, contact with vomitus particles, consumption of contaminated food or water or through contact with contaminated surfaces [58]. Patients infected with norovirus may shed large numbers of noroviruses for protracted periods even after the symptoms resolution and may act as a source for nosocomial transmission [59]. Shellfish and frozen raspberries have served as vehicles for the transmission of noroviruses as a result of being contaminated with human fecal material or through using sewage-contaminated water for irrigation or by contact with infected food handlers during harvesting and processing [60,61]. Norovirus can be harbored by a wide range of hosts including humans, canines, sheep, cattle, pigs, rodents, bats and felines [35,41]. Although some serological studies suggested the possible transmission of animal-derived norovirus to humans, there is no evidence that animal norovirus can infect humans [35,62]. Norovirus transmission can be airborne and can also take place via fomites, which have the potential to spread the virus and magnify the size of illness outbreaks [46]. The CDC suggested that norovirus may spread through the air where the small droplets of oral discharge from an infected person contact surfaces or are inhaled by a healthy person [63]. Norovirus RNA was detected in 24% (21/86) of air samples from rooms housing 10 patients [64]. Recently, the fomite transmission of norovirus was detected by re-analyzing the transmission routes of a previously reported hotel restaurant outbreak. The results showed that the attack rate distribution matched well with that of the infection risk via the fomite route [65]. In another study, it was confirmed that the fomite-mediated exposures significantly contributed to large portions the of attack rates in outbreaks with multiple transmission modes [66]. 

Leafy greens, fresh fruits and shellfish are the foods commonly involved in norovirus outbreaks [67]. Culinary herbs, lettuce (romaine, iceberg, mesclun), green onions, strawberries and deli ham have also been implicated in norovirus outbreaks [68]. Food plant workers can spread the virus when touching ready-to-eat foods with their bare, inadequately washed hands [67]. Of the different means for transmission of norovirus in food, the most common setting was eating out (37%). Others include: open-headed lettuce during retail sale (30%), takeaway foodservice (26%), raspberries at retail sale (4%), and oysters during retail sale (3%) [31]. Norovirus is also associated with outbreaks in restaurants, schools, cruise ships, and with healthcare situations including in hospitals [69].

Noroviruses are thought to possess different mechanisms to circumvent and antagonize host immune responses. This necessarily leads to lengthy norovirus infections and subsequent protracted viral shedding. Immune system antagonistic activity results from the actions of specific nonstructural norovirus proteins such as p22 and p48, which interfere with functional protein transferring and cellular secretory pathways. Subsequently, the Golgi apparatus can be disassembled, and this interferes with the ability of the infected cell to develop an effective immune response [70]. Additionally, impairing protein transfer reduced the secretion of cytokines [71]. Further, it has been reported [70] that norovirus protein VF1 antagonizes the expression of antiviral genes, and that norovirus protein VP2 restricts antigen presentation and overall protective immunity induction.

It is notable that noroviruses showed great survivability on food surfaces. In human norovirus genogroup II, genotype 4 virus-like particles were able to attach to lettuce leaf surfaces and cut edges [72]. It has been demonstrated that the virus–lettuce attachment is due to the presence of Histo-Blood Group Antigen (HBGA)-like carbohydrates on lettuce leaves and the ability of norovirus to bind the exposed fucose moiety in the cell wall of lettuce [73]. This attachment was also mediated through the viruses’ HBGA binding sites [74]. In another study, murine norovirus, a virus genetically more closely related to human norovirus, persisted on lettuce for 14 d at 4 °C and for 3 d at room temperature [75]. Furthermore, human norovirus transported from the roots of lettuce and spinach to the leaves were internalized in the leaves and remained stable within leaves and roots at similar inoculated RNA titers for 6 d [76]. The recovery and adhesion of noroviruses on foods is affected by the physicochemical parameters of food. For example, the recovery of infectious norovirus particles from turkey was 68% compared to 9.4% from strawberries [77]. A murine model norovirus persisted well and remained in an infective state, with only a 1.29–2.28 log decline in infectivity from 10^5^ plaque forming unit (PFU) inoculated on food contact surfaces made of ceramic, glass, plastic rubber, stainless steel, and wood for 28 d at room temperature [78].

Noroviruses were found to be resistant to freezing and to relatively high temperatures up to 60 °C and have even been detected in steamed shellfish. Also, noroviruses may persist in chlorine solution up to 10 ppm. However, practices such as minimizing the handling of foods, using disposable gloves, chilling cooked food, and the proper, frequent washing of hands were advised to substantially reduce the foodborne transmission of noroviruses [79]. Some non-thermal strategies including cold atmospheric plasma, irradiation, ultra violet light, and high hydrostatic pressure can be used to inhibit noroviruses in foods [80]. 

### 2.2. Rotavirus

Rotaviruses are a genus of positive-sense, double-stranded (ds)RNA viruses belonging to the family *Reoviridae*. Since their discovery in 1973, rotaviruses have become known as the leading cause of severe childhood diarrhea worldwide [81,82,83]. Rotaviruses are organized into nine species: A to J [81,82]. There are four specific subgroups within group A. Groups A, B, C and H are the major groups that infect humans and animals, and, of these, group A is predominant, while strains that belong to species D, F, and G mainly infect animals [81,82].

Epidemiologically, rotavirus A has the highest clinical impact in humans, as it causes the most severe gastroenteritis among children, worldwide, compared to the other groups [38,81,84,85]. However, rotavirus group B has been found responsible for adult and child diarrheal illness in China [40]. Group C has also been linked to sporadic cases of acute diarrhea among humans, worldwide. Zhao et al. (2022) reported that the infection rates of rotavirus C in humans has decreased from 3% before 2009 to 1%, whereas the infection rates in animals increased from 10% to 25% [86]. 

The mortality rate of rotavirus infection is 0.3–1.8% [81,87]. In general, adults’ symptoms tend to be milder than infants’ and young children’s because immunity is developed with previous infections [88]. The general and clinical characteristics of rotavirus are presented in Table 1.

The virus is mainly spread via the fecal–oral route [84], and upon ingestion the virus primarily attacks the enterocytes of the small intestine villi. A usual cycle of viral replication results in the compromise of the enterocyte cell function, leading to the inadequate absorption of nutrients, fluids and electrolytes. The subsequent replication of secretory crypt cells results in fluid and electrolyte accumulation in the gut lumen. Moreover, compromised enterocyte function results in reduced digestive enzyme expression, which causes sugars to be concentrated in the gut lumen. The above two developments result in the classical clinical symptoms of rotavirus-associated diarrhea [81,87,89,90]. 

A low dose of rotavirus can be infectious, and it has been reported that one plaque forming unit (PFU) is sufficient to cause infection in humans [91]. Rotavirus exhibits considerable environmental stability, which allows it to persist in fresh water and foods, even when exposed to light, which can inactivate other enteric RNA viruses. This resistance is due to its presence as highly persistent viral vesicles which can also transmit more than 25 virions to the host cell at once [28]. This facilitates the development of illness outbreaks following the consumption of contaminated water or ice and foods including seafood and salads [88,92].

Rotavirus can persist well at refrigeration temperatures, and can also persist in soil for more than a week, even at temperatures up to 37 °C. In contrast, sand has been observed to decrease the infectivity of the virus. Additionally, following a temperature change from 4 to 37 °C, viral populations have also been observed to decrease. This is an encouraging observation, as it indicates that the virus may not be very thermostable [93]. Bovine rotavirus was observed to decrease in infectivity by up to 8 log on stainless steel surfaces when stored for 21 d at 21 °C. However, it did not lose infectivity on blueberries or in bottled water even up to 21 d at 4 °C or −20 °C [94]. In juices, rotavirus was observed to persist well up to 3 h in papaya and honeydew melon juices at room temperature. However, viral infectivity decreased linearly within 1 h in pineapple juice stored at the same temperature. The acidity and other pineapple juice constituents may explain this observation [95]. 

### 2.3. Sapovirus

Sapovirus was first recognized during a large outbreak in 1977 in Sapporo, Japan. Sapovirus is a genus of non-enveloped, positive-sense ssRNA viruses belonging to the family *Caliciviridae* [42]. Sapovirus are classified into 19 genogroups (GI through GXIX), where GI, GII, GIV and GV are specific to humans, while other genogroups infect animals including bats, sea lions, dogs, pigs, minks and rats [96,97]. Sapoviruses are enteric viruses that cause gastroenteritis in both developed and developing countries, affecting people of all age groups including infants and children [98]. According to systematic research by Magwalivha et al. (2018), who examined 45 sapovirus prevalence studies published between 2004 and 2017 in 19 low-and middle-income countries, the overall prevalence of sapovirus was 6.5%, with a significantly higher frequency in lower-income countries [99]. Most these studies (78.6%) investigated the prevalence of sapovirus in children, and the sapoviruses GI and GII were most dominant [99].

Sapovirus infections follow a seasonal pattern, with most cases occurring during the winter months [58,100,101]. Tang et al. (2022) found that sapovirus significantly contributed to the overall burden of diarrhea in Chongqing, China, particularly in children < 4 y old, with sapoviruses GI and GII being most frequently detected [101]. Similarly, sapovirus genotypes I and II were detected in 3.5% of 742 stool specimens from children < 5 y hospitalized with viral etiologies in southwestern India [102]. In the US, sapovirus was identified in 10% of children < 18 y who were receiving care for diarrhea in both outpatient and inpatient settings [103]. Similarly, sapovirus was responsible for 13% of the gastroenteritis outbreaks reported in north–east England from July 2016 to July 2018 [104]. Sapovirus was detected in 24.7% of diarrheal stools collected from children ≤ 24 months old in eight countries including Brazil, Bangladesh, Peru, Pakistan, Tanzania, South Africa, India and Nepal [105].

The viral capsid proteins of sapovirus play a crucial role in its attachment and entry into host cells. Furthermore, the replication of sapovirus within the epithelial cells lining the small intestines lead to inflammation and the characteristic symptoms of gastroenteritis [97]. The clinical and epidemiological characteristics of sapovirus show similarities with norovirus. However, sapovirus causes a lower number of foodborne outbreaks and illnesses [106,107]. Occasionally, the symptoms of sapovirus infection are followed by dehydration, malnutrition, secondary infection, and finally hospitalization in severe cases, particularly among infants, young children, and immunocompromised individuals [108,109]. The general and clinical characteristics of sapoviruses are presented in Table 1. 

Sapovirus is usually transmitted through the fecal–oral route, and when the virus is shed in the feces of an infected individual it can be transmitted to others if they come in contact with contaminated surfaces, food, or water [10]. Sapovirus has been detected in water, untreated and treated sewage and shellfish including oysters and clams [10,110]. Moreover, sapoviruses have been associated with outbreaks linked to the consumption of raw or undercooked shellfish. These can become contaminated if they are harvested from waters contaminated with human sewage [107]. The virus can also spread from person-to-person via close contact (hospitals, nursing homes, child care centers and schools), while caring for an infected individual, or sharing contaminated personal items [111]. In addition, contacting contaminated surfaces and then touching the nose, eyes or mouth can result in transmission of sapovirus [111]. 

Sapovirus can persist on surfaces for a significant amount of time, depending on the environmental conditions. For example, the virus can persist for days to weeks on hard surfaces, such as stainless steel and plastic, especially in cool and damp environments [112]. Esseili et al. (2015) reported that porcine sapovirus persisted on spinach and lettuce leaves for 7 d at 4 °C, and the phytopathogen, *Xanthomonas campestris* pv. *vitians* 701a, promoted sapovirus persistence on lettuce [113]. In another study, sapovirus persisted on lettuce for up to 3 d at room temperature or for 14 d at 4 °C [75]. 

### 2.4. Astrovirus

Astroviruses are a group of positive-sense, ssRNA viruses belonging to the family *Astroviridae*, genus *Mamastrovirus*, with four species affecting humans: 1, 6, 8, and 9 [44]. Mamastrovirus-1 includes eight genotypes of classical human astrovirus (human astrovirus -1 to -8) known to cause gastroenteritis in humans. Mamastrovirus-6 has the Melbourne (MLB) clade and includes novel MLB-1, -2, and -3 genotypes. Mamastrovirus-8 clade includes the novel Virginia/Human-Mink-ovine-like (VA/HMO) -2, -4, and -5 genotypes. Mamastrovirus-9 clade includes the Virginia/Human-Mink-ovine-like (VA/HMO)-1 and -3 genotypes [114]. Recently, a receptor was identified for Mamastrovirus-1 called the neonatal Fc receptor (FcRn) which is a functional receptor for human astroviruses [115]. Reviewing the recently published literature on astrovirus infections has revealed infections characterized by gastroenteritis from acute inflammation commonly affecting school age children (<5 y) during spring and autumn, with differences in the prevalence of genotypes in different geographical areas. Further, individuals infected with one strain do not acquire immunity against other strains. Globally, human astrovirus-1 and MLB-1 were the predominant genotypes detected in gastrointestinal tract infections [116,117,118,119]. Additionally, the most frequent viral co-infections reported with astrovirus illness involved norovirus and rotaviruses [120].

Astroviruses are spread worldwide, and they are accountable for 2–9% of acute, non-bacterial diarrheal illnesses in children, even though their occurrence in clinical specimens was higher and reached 61% in symptomatic and asymptomatic individuals [116,118,121]. In other works, it was found that human astrovirus causes 10% of acute gastroenteritis sporadic cases in children of <3 y, and that most outbreaks occurred mainly in healthcare and daycare centers, while in some developing countries, infection rates reached 20% [117]. The symptoms of gastrointestinal tract and inflammatory lesions affecting the meninges and brain tissue as encephalitis and meningitis were also reported [122,123]. The general and clinical characteristics of astrovirus are presented in Table 1.

Astroviruses, although less frequently reported in gastrointestinal tract infections than noroviruses, were detected in symptomatic and asymptomatic cases [124]. Water and food contamination routes are most commonly reported for the transmission of astroviruses with a relatively low infectious dose [10,116]. Persistence time investigations showed that the virus persists for two months at cold temperatures on surfaces, and this was relatively shorter than rotaviruses or noroviruses [125]. Human astroviruses can infect the epithelium of the duodenum, and have been detected in children’s stool. The virus was also found in the feces of some animals (e.g., cattle, sheep, poultry, deer, cats, dogs, rats and bats) [67]. Astroviruses are transmitted mainly through the fecal–oral route, either by the ingestion of contaminated water and food or direct contact and cause human infections when present in a relatively low dose [10]. Most astrovirus foodborne illnesses resulted from foods contaminated by infected food handlers, or the consumption of contaminated shellfish or produce originally grown or irrigated with contaminated water [126]. Recently, some reported foodborne outbreaks were linked to the consumption of bivalve mollusks contaminated by polluted water [10].

### 2.5. Adenovirus

Adenovirus was first identified in 1953, isolated from human adenoid tissue. Adenoviruses are non-enveloped viruses containing an icosahedral nucleocapsid with a dsDNA, and they belong to the family *Adenoviridae* and the genus *Mastadenovirus* [46]. The family *Adenoviridae* contains five genera: *Atadenovirus* (infects sheep, cattle, ducks and possum); *Aviadenovirus* (infects birds); *Ichtadenovirus* (infects sturgeon); *Mastedenovirus* (infects mammals), and *Siadenovirus* (infects reptiles and birds) [6]. There are 51 serotypes of human adenovirus which are further divided into seven subgroups (A–G). Adenovirus can infect and reproduce in the respiratory tract, GI tract epithelial cells, urinary bladder, and eyes. Adenovirus may cause hidden infection in lymphoid cells and lytic infection in epithelial cells [127]. Human adenovirus serotypes 40 and 41 in group F are the major causes of gastroenteritis in young children [46,128]. Recent reports showed that a remarkably high burden of gastroenteritis among children in low- and middle-income countries was caused by adenovirus serotypes 40 and 41 [128]. The incidence of adenovirus serotypes 40 and 41 infections in children with diarrhea was 13% in Guatemala [129], 5.1% in Nigeria [130], and 1.5% in Brazil [131].

Other serotypes might also attack the upper respiratory tract or eyes [132]. Although adenovirus infection is rarely associated with serious illnesses and deaths, patients with respiratory or cardiac diseases, immunocompromised individuals, or infants are at higher risk of developing severe illnesses [10,133]. The general and clinical characteristics of adenovirus are presented in Table 1.

Adenoviruses are spread via aerosolized droplets, blood, and the fecal–oral route [134]. In addition to these, the surfaces of objects or materials (fomites) play an important role in the transmission of adenovirus [135]. The incubation period of adenovirus depends on the viral serotype and transmission mechanism, and may range from two days to two weeks [134]. Clinical symptoms often occur in children; however, infected adults are asymptomatic. Common symptoms associated with adenovirus infection include gastroenteritis, conjunctivitis, acute respiratory illness and fever [136]. Uncommon symptoms include bladder inflammation/infection and neurological complications [137]. The treatment of adenovirus infection is not required in most cases, and there is no specific treatment or approved antiviral medicine; however, in severe cases, hospitalization and rehydration may be required [138]. Outbreaks of adenovirus have been reported globally in closed or crowded settings, such as dormitories, healthcare facilities, and among military recruits [136], since adenovirus transmission is facilitated in congregate environments [139]. Waterborne outbreaks of human adenovirus have been found to be associated particularly with swimming pools, and clinical findings involve conjunctivitis [46]. Adenoviruses have been reported in wastewater, sludge, shellfish, and marine, drinking, and surface waters [6]. 

Compared to other enteric viruses, adenovirus can persist better in wastewater and can remain infectious for extended periods of time in untreated waters. The high rate of and prolonged adenovirus shedding by infected individuals suggests that adenovirus can spoil surface water resources via contaminated domestic wastewater [140]. Adenoviruses were detected in water and shellfish samples collected from a number of coastal oyster breeding farms and fishing ports in Taiwan during 2016–2017. The primary source of this viral contamination stemmed from the direct discharge of wastewater from livestock farms, domestic sewage, and fish markets into the coastal environment [141]. Adenovirus can persist on inanimate objects for an extended period, sometimes for several weeks following contamination [142]. Adenoviruses are resistant to UV light, which causes damage to DNA without affecting the proteins that are associated with adenoviruses’ capacity to infect and replicate [143]. Adenovirus is often resistant to many lipid disinfectants due to its non-enveloped nature; however, it is inactivated by formaldehyde, bleach and heat [134]. In swimming pools, chlorine levels must be adequate, as chlorination failures are often a major factor contributing to illness outbreaks [46].

### 2.6. Hepatitis A Virus

Hepatitis A virus is a tiny non-enveloped member of the *Hepatovirus* genus within the *Picornaviridae* family. It contains positive-sense, single-stranded RNA that exhibits substantial genetic variability worldwide [144]. Like norovirus, it is often transmitted via the fecal–oral route. However, recent studies reported that hepatitis A virus is released from liver host cells and is capable of circulating in the bloodstream as membrane-cloaked ‘quasi-enveloped’ virions [53,57]. Human hepatitis A virus is grouped into several genotypes including I, II, and III, which are subdivided into six sub-genotypes, named IA, IB, IIA, IIB, IIIA, and IIIB [144].

Hepatitis A virus is one of the most well-studied foodborne viruses. The virus was identified for the first time in the feces of hepatitis A patients in 1973 [145]. Hepatitis A illness is a public health problem involving 1.4 million detectable cases worldwide, yielding an estimated 15,000 to 30,000 deaths per year [146,147]. However, the incidence rate of hepatitis A infection is underestimated due to the self-limited nature of the disease, and it is estimated that 100 million individuals are infected annually [147]. According to the Global Burden of Disease data, the worldwide incidence of hepatitis A infections raised by 13.9% to reach 158.9 million in 2019 compared to 139.5 million in 1990 [148]. The prevalence of hepatitis A infection in many countries is variable and dependent upon the level of hygiene practiced. As a result, the infection is common and endemic in several regions of the world including Africa, Central and South America, Asia, and the Western Pacific region [149]. In the US, 12,474 cases of acute hepatitis A were documented by the CDC in 2018; however, the widespread use of childhood hepatitis A immunization has reduced the prevalence of hepatitis A illness [149]. In contrast, the average rate of severe hepatitis A-associated hospitalization and hepatitis A-related deaths have increased recently [149].

Hepatitis A virus usually causes acute, asymptomatic, self-limited hepatitis [47]. These infections are most often contracted by very young children [150,151]. The general and clinical characteristics of the hepatitis A virus are listed in Table 1. In less than 1% of human cases, hepatitis A infection can progress to severe liver disease [47]. The mortality rate associated with acute hepatitis A infection in children and adults aged < 50 y ranges from 0.3% to 0.6%, whereas the mortality rate associated with acute hepatitis A infection in adults older than 50 y ranges from 0.8 to 5.4%. Hepatitis A infection usually confers lifelong immunity, and it is a vaccine-preventable disease [152]. Several internationally available vaccines are safe and effectively provide good levels of protection with minimal side effects [153].

The most common manner of hepatitis A transmission is through the fecal–oral route and involves the ingestion of contaminated water or food and by direct contact with infected individuals [47]. It has been reported that food products contaminated with hepatitis A virus are responsible for 2–7% of all worldwide hepatitis A illness outbreaks [154]. Rarely, cases of hepatitis A infection have been reported to occur following blood transfusion or organ transplantation [146,149].

Hepatitis A virus infection has been associated with the consumption of contaminated raw shellfish, fruits, vegetables, ready-to-eat foods, and water [47,155]. The consumption of raw or undercooked shellfish grown in contaminated water is the major reason for hepatitis A virus outbreaks [47,156]. The incubation period of hepatitis A virus ranges between 15–50 d with a mean of 28 d [156].

Hepatitis A virus is highly resistant to environmental stresses because it has a non-enveloped highly cohesive capsid [157]. Foods can be contaminated with hepatitis A virus at any point in the food chain, and the virus can persist for months in food and related environments (on inanimate surfaces, water, soil, bivalve mollusks and sediments). It is more resistant than bacteria to commonly used control interventions including heat, radiation, disinfection, refrigeration, freezing, pH and high-pressure processing [47]. The virus can persist for several hours or even days on human hands and environmental surfaces indoors, respectively [158]. Additionally, hepatitis A virus can remain intact and infectious under freezing conditions, and it can remain in salt or fresh water for 12 months [159]. The large numbers of hepatitis A virus particles in feces and the extended incubation period significantly contribute to the occurrence of hepatitis A illness outbreaks, mainly those linked to food handlers [47,160].

### 2.7. Hepatitis E Virus

Hepatitis E virus is a positive-sense, single-stranded RNA virus, and it is classified into at least eight genotypes (1–8) based on genetic differences, and it belongs to the family *Hepeviridae*. Genotypes 1 and 2 primarily infect humans and are linked to large outbreaks in developing countries. Genotypes 3 and 4 infect both humans and animals, with pigs being the primary reservoir. Genotypes 3 and 4 are responsible for most cases of hepatitis E in developed countries [161,162]. Similar to hepatitis A virus, hepatitis E virus was also considered non-enveloped. However, recent studies designated the virus as ‘quasi-enveloped’ [53,57]. The virulence factors of hepatitis E virus are not well understood, but Smith et al. (2014) found that specific mutations in the viral genome may contribute to differences in pathogenicity and clinical outcomes [163].

The clinical presentation of hepatitis E infection can vary from mild to severe. Most infections are asymptomatic or cause a self-limiting acute hepatitis. However, in certain populations, such as individuals with pre-existing liver disease and pregnant women, hepatitis E infection can lead to fulminant hepatitis and increased mortality [164,165,166]. Chronic hepatitis E, characterized by persistent viremia and liver inflammation, has been observed in immunocompromised individuals, including HIV-infected patients and organ transplant recipients [6,167]. The general and common clinical characteristics of the hepatitis E virus are presented in Table 1. 

The epidemiology of hepatitis E virus varies across different regions. In developing countries, hepatitis E virus is primarily transmitted through contaminated water, leading to large outbreaks. In these areas, hepatitis E illness is most commonly associated with genotypes 1 and 2 [168]. In developed countries, sporadic cases of hepatitis E virus are more common, with genotypes 3 and 4 prevalent. Recent studies have shown an increasing number of autochthonous (locally acquired) cases in developed countries due to zoonotic transmission. These cases are predominantly associated with genotypes 3 and 4 [169].

Hepatitis E virus can spread via several routes, including the fecal–oral route, in regions with contaminated water and water supplies of insufficient sanitation. The consumption of contaminated water or food, mainly raw or undercooked shellfish, is linked to hepatitis E infection. In developed countries, zoonotic transmission is a significant concern, primarily through the consumption of undercooked or raw pork products. Additionally, hepatitis E virus can be transmitted through blood transfusion or following organ transplantation, although these routes account for a small proportion of cases [6,170].

Meat products from hepatitis E virus-infected animals may transmit the virus to humans [171]. Replicative hepatitis E virus was detected primarily in the liver of infected animals, and also in the gastrointestinal tissues, mesenteric and hepatic lymph nodes, and the spleen [171,172]. Furthermore, after inoculating pigs intravenously, hepatitis E virus has been recovered from their salivary glands, tonsils, lungs, stomach, kidneys, and multiple muscle masses [171]. In addition to pigs, some other animal species also serve as potential reservoirs for hepatitis E virus, including rats, rabbits, chickens, ferrets, wild boar, domestic swine, mongoose, cutthroat trout, bats, and deer [173].

Studies have demonstrated the stability of hepatitis E virus in water, including both freshwater and seawater, for several weeks [174]. Hepatitis E virus has also been detected on surfaces such as stainless steel and plastic, although the length of its persistence may vary depending on environmental conditions [174]. The virus can persist in pork meat for extended periods, especially when stored at low temperatures.

## 3. Other Potential Emerging Viruses

Different definitions have been suggested for the term ‘emerging pathogens’. In this review, “emerging foodborne viruses” include viruses that have been known as pathogens but have only recently been shown to be transmitted via foods [175]. Recently, several new viruses were isolated from food products, and these might be considered emerging foodborne pathogens, which is alarming because of the potential risk for transmission to humans through the food chain. Zoonotic viruses including nipah viruses, ebola viruses, avian influenza viruses, aichi virus, tick-borne encephalitis virus, and coronaviruses (SARS-CoV-1, SARS-CoV-2 and MERS CoV) may have the potential to be transmitted to humans and cause gastroenteritis via the consumption of contaminated foods, particularly raw or undercooked meat products from infected animals [67,138,176]. However, the reliable detection of viruses in food products is a challenge due to the absence of viral culture methods, variable effectiveness of detection methods, low levels of contamination, the presence of inhibitors, and the heterogeneity of viral distribution in foods [11,177]. It worth mentioning that the detection of the nucleic acid of these emerging viruses in food products using polymerase chain reaction (PCR) and other techniques says nothing about their infectivity, since it does not distinguish between viral genome particles and viral infectivity. The detection results are varied based on the food products, the virus distribution in the food matrix, and the presence of PCR inhibitors [178,179]. Indeed, numerous studies have determined that even under ideal conditions, the particle-to-PFU ratios of many animal viruses are in the range of hundreds to one and may be as high as thousands to one. This particle-to-PFU ratio likely increases when viral particles are subjected to conditions that may affect infectivity without destroying the physical particle. Therefore, experimental methods for the inoculation of viruses in animals or on cell culture techniques are required to determine the infectivity of viruses detected in food samples [178,179]. Furthermore, it is difficult to demonstrate a direct epidemiological correlation between the detection of emerging viruses’ genomes in food and the occurrence of foodborne outbreaks. Yet, the detection of the genomes of these viruses in food should be taken as a sign of potential risk.

### 3.1. Tick-Borne Encephalitis Virus 

Tick-borne encephalitis virus (TBEV) is an enveloped, positive-sense, ssRNA virus that belongs to the genus *Flavivirus* and family *Flaviviridae*. It is a zoonotic virus transmitted to sheep, goats, and cows via ticks. The virus was detected in several dairy products including milk and cheese. In Poland, the RNA of tick-borne encephalitis virus was detected in raw milk samples from 7 of 63 cows (11%), 6 of 29 goats (21%), and 6 of 27 sheep (22%) [180]. In Norway, the virus was detected in 6 of 112 (5%) cow milk samples [181]. The virus was also detected in 5 samples out of 22 (22%) different types of cheeses including soft, cream, and ripened cheeses [182]. Tick-borne encephalitis virus spread to humans by ingesting unpasteurized dairy products from infected animals. There were several tick-borne encephalitis foodborne outbreaks reported in the EU [183,184]. The number of infections of tick-borne encephalitis virus has increased in the EU over the last decade [183,185]. Therefore, preventive strategies including the pasteurization of milk and TBEV vaccines are available and can be used to prevent illness from this virus.

### 3.2. Nipah Virus

Nipah virus is a negative-sense ssRNA virus belonging to the family *Paramyxoviridae* and the genus *Henipavirus*. The fruit bat is the main host for the virus, which causes severe respiratory and neurological illnesses with a high mortality rate [186]. Nipah virus was first recognized in Malaysia and Singapore in 1998–1999 after a large outbreak with 283 illnesses and 109 deaths [187]. It was suggested that fresh date palm sap was linked to some cases of infection where the nipah virus may have been transmitted to humans from fruit bats that drank at night from the clay pots used to collect the sap [188]. A subsequent study proved that the nipah virus was able to infect Syrian hamsters which drank palm sap containing the virus [189]. Recently, in 2018, a nipah virus outbreak with 23 cases and a case-fatality rate of 91% was reported in the Kozhikode district of Kerala, a South Indian state, and it was suspected that the patients had contracted nipah virus from fruit-eating bats [190].

### 3.3. Ebola Virus

Ebola viruses are negative-sense filamentous ssRNA viruses that belong the genus *Ebolavirus* and the family *Filoviridae*. Ebola virus causes severe human and animal illnesses. It was first revealed in 1976 in the Democratic Republic of Congo after a fatal outbreak occurred. The average ebola virus disease case fatality rate is around 50% [191]. Ebola virus probably spreads by the transfusion of blood or other body fluids of infected persons and via person-to-person contact [192]. Following the discovery of ebola virus in bushmeat, which is raw or minimally processed meat from wild animals, bushmeat was considered a vehicle for ebola virus outbreaks in humans. The virus also can be directly spread from one person to another [193,194].

### 3.4. Avian Influenza Virus

Avian influenza viruses are a group of viruses that have enveloped negative-sense, ssRNA with segmented genomes and belong to the genus *Alphainfluenzavirus* and the family *Orthomyxoviridae* [33]. Different samples from live birds including chicken and ducks as well as their meats were positive for the infectious H5N1 avian influenza virus [195,196,197]. Although no foodborne outbreaks related to the avian influenza virus have been reported, some experiments showed that the consumption of duck blood transmitted the virus to carnivorous animals such as tigers, leopards, and domestic dogs and cats. This suggests that consumption of contaminated products may be responsible for H5N1 avian influenza virus infection in humans [197]. In addition, the CDC stated that all reported infected cases with avian influenza virus had a recent exposure to sick or dead poultry. However, people who are in direct contact or have recreational exposures to infected poultry may be at high infection risk. Moreover, direct human-to-human transmission mostly occurs as a result of family or healthcare worker exposure [198].

### 3.5. Aichi Virus 

Aichi virus is a small non-enveloped positive-sense, ssRNA virus in the genus Kobuvirus in the family Picornaviridae with positive-sense and icosahedral morphology. It was first detected in 1989 in the stool samples of patients with gastroenteritis following the consumption of oysters in Aichi, Japan [199,200]. The virus was also isolated from the *vero* cells of 12.3% (6/47) patients from different gastroenteritis outbreaks, and 2.3% (5/222) of Pakistani children with gastroenteritis [201,202]. Le Guyader et al. (2008) reported in a retrospective study performed in France among children between 2001 and 2004 that 0.9% of the collected stool samples were positive for aichi virus [203]. Aichi virus was isolated from the fecal specimens of patients involved in an acute gastroenteritis outbreak in Germany [204]. Moreover, Aichi virus was detected in the randomly selected stool specimens of children with diarrhea in Brazil [204]. Therefore, it has been anticipated to be the cause of human gastroenteritis with the potential for transmission via the fecal–oral route by contaminated food or water [10]. Furthermore, aichi virus was detected in 6.6% (4 of 60) shellfish samples collected in Tunisia [205]. More recently, aichi virus was detected in 3/170 (1.8%) of retail shellfish including oysters and mussels in the Apulia region of Italy [206].

### 3.6. Coronaviruses (SARS-CoV-1, SARS-CoV-2 and MERSCoV)

Coronaviruses are group of enveloped viruses with a positive-sense ssRNA genome belonging to the family *Coronaviridae*. Coronaviruses cause illnesses mainly in mammals and birds. These viruses may also cause human respiratory tract infections ranging from mild to lethal disease [207]. During the period 2002–2004, an outbreak of a new disease of severe acute respiratory syndrome (SARS) involving more than 8000 people with 774 deaths caused by coronavirus was reported in 29 countries [208]. Another outbreak linked with a different coronavirus, Middle East respiratory syndrome coronavirus (MERS-CoV), was first identified in 2012 in the Middle East region and expanded to reach 27 countries where it infected more than 2600 individuals and caused 880 deaths [209]. 

The global coronavirus 2019 (COVID-19) pandemic caused by severe acute respiratory syndrome coronavirus 2 (SARS-CoV-2) has diseased > 772 million individuals with 6.99 million deaths [210]. 

Several studies showed that coronaviruses can persist on different foods types such as milk, fresh produce (blueberries, strawberries, apples, avocado shells and pulp, grapes, mushrooms, spinach, and tomatoes), seafood (salmon, oyster and shrimp), ready-to-eat deli food products (cheese, salami and roasted turkey), and meat and poultry products (chicken, beef, plant-based meat alternative and pork) during storage in refrigerators (4 °C) and freezers (−10 to −80 °C) [211,212,213,214,215,216,217,218]. Coronaviruses may also persist on stainless steel and plastic food contact surfaces for up to 72 h [217,218]. In another study, SARS-CoV-2 persisted on plastic wrap, fruit wax, and cardboard takeout container surfaces kept for 7 d at 4 or 20 °C and a relative humidity of 30–70%. However, the half-life for the infectious virus was ~24 h at 4 °C and ~8 h at 20 °C on all surfaces tested. In addition, the genomic material of SARS-CoV-2 was detected at both temperatures up to 7 d with negligible to no loss compared to the initial inoculum [219].

Furthermore, it was suggested that SARS-CoV-2 identified in frozen food products and food packaging may be capable of initiating illnesses after the cold-chain distribution of contaminated food products [220,221]. In a large survey in China, SARS-CoV-2 was detected in 1455 frozen food-related samples (1398 samples of foods/food packaging and 52 samples of storage environments) out of the 55.83 million samples collected [222]. However, SARS-CoV-2 was only detected in one sample (automated teller machine) out of 2055 surfaces in public spaces and food surfaces in Lima, Peru [223]. Furthermore, SARS-CoV-2 was not detected in 9354 cold-chain food-related environmental samples (4708 dining utensils, 1933 supermarket environment, 1543 food freezers or fish tanks, 1032 fire or sewer hydrants, and 138 clothing batches of food workers) collected in Xiangyang in China. Meanwhile, the virus was extracted from two samples among 23,187 various cold-chain foods (13,859 meats, 3483 seafoods, 1046 fruits, 2973 fresh water samples, 164 vegetables, and 1661 refrigerated drinks) sold in different business premises [224]. In another work, 957 surface samples at food retail stores in Ontario, Canada were negative for SARS-CoV-2 [225]. These viruses are zoonotic pathogens that can be spread from animals to humans. Although WHO, USFDA, and EFSA suggested that food products are unlikely to serve as sources or routes of SARS-CoV-2 transmission and currently there is no evidence that food products are associated with COVID-19 illnesses, some reports indicated that foods contaminated with these viruses have the potential to cause illnesses [211,212]. 

## 4. Viral Foodborne Outbreaks and Illnesses

In recent years, many viral foodborne outbreaks have been documented worldwide. According to the U.S. Food and Drug administration (FDA), an outbreak occurs when two or more people eat or drink the same contaminated food or water, respectively. In general, viruses have low infectious doses with around 100 infectious viral particles or fewer able to cause disease. In addition, their level of virulence can lead to large outbreaks in a relatively short time. These make viral foodborne illness outbreaks very different from bacterial outbreaks, although with foodborne viruses the mortality rate is lower than with bacteria including *Listeria monocytogenes*, hemorrhagic *Escherichia coli*, and *Clostridium botulinum* [226,227].

Epidemiological outbreak reports in the last decade have indicated that enteric viruses, particularly noroviruses, were the foremost cause of foodborne illness in developed regions [228,229,230]. Other enteric viruses, including rotavirus, hepatitis E virus, sapovirus, astrovirus, adenovirus and hepatitis A virus were also linked to foodborne illnesses, and they can be transmitted via contaminated foods [138,229]. 

**Figure 1:**
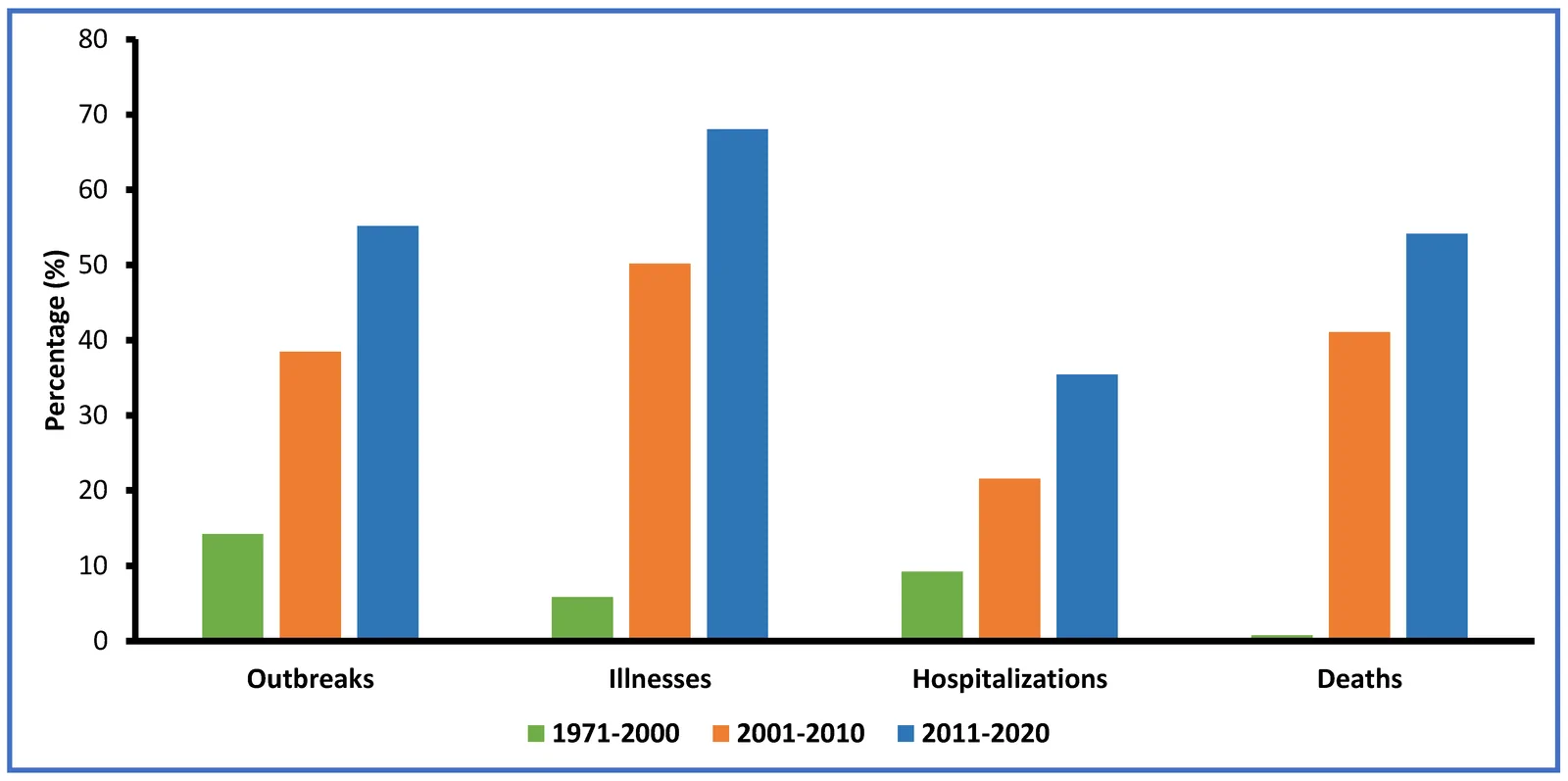
Percentages of viral outbreaks, illnesses, hospitalizations and deaths reported in the US during 1970–2020 (data extracted from CDC, 2022) [228].

Scallan et al. (2011) expected that viruses are responsible for about 59% of foodborne cases in the US. According to CDC data during 1970–2020, 57,649 foodborne outbreaks with more than 2 million illnesses and 2205 deaths were reported in the US. Viruses were responsible for 28,214 (49%) outbreaks involving 0.92 million (45%) illnesses with 990 (45%) deaths [2]. Furthermore, viruses were responsible for 55, 68, 35 and 54% of the total outbreaks, illnesses, hospitalizations and deaths, respectively, that occurred during 2010–2020 (Figure 1). Among viral outbreaks, norovirus was linked to 27,621 outbreaks with 0.9 million illnesses and 957 deaths, followed by sapovirus (199 outbreaks with 8172 illnesses and 3 deaths), rotavirus (199 outbreaks with 6237 illnesses and 20 deaths), and viral hepatitis (143 outbreaks with 3945 illnesses and 11 deaths). Adenovirus, astrovirus, and other viruses were also linked to outbreaks (Table 2).

In Canada, norovirus is recognized as the pathogen that caused most hospitalizations and was the second highest cause of deaths [232,233]. In 2021, about 4005 foodborne illness outbreaks were reported in the European Union (EU), with norovirus and other caliciviruses being the third most regularly documented agent causing foodborne outbreaks. These viruses were the foremost causative agents in Sweden, Finland, Denmark, Latvia, Czechia, and Belgium. France attributed 112 (43.6%) outbreaks to norovirus and other caliciviruses. Furthermore, norovirus and other calicivirus were the most frequent agents linked to foodborne outbreaks associated with crustaceans, shellfish, mollusks and other fish products in the EU [234]. Hashemi et al. (2023) reported that the incidence rates of norovirus foodborne illnesses ranged from 418–9,200,000 in the US and Europe and from 11–2643 cases in Asia [235].

Norovirus is the leading cause of acute gastroenteritis outbreaks and has the highest illnesses burden in many countries including the US, Australia, the UK, the Netherlands, New Zealand, and France [2,34,232,236,237,238]. It is estimated that one in five acute gastroenteritis cases is infected with norovirus, and this represents a total of 685 million cases. Children aged < 5 years account for about 200 million cases, which means noroviruses cause approximately 50,000 deaths in children each year, commonly in developing nations. However, norovirus illness happens in both developed and developing countries, where together it is expected that the global cost of these illnesses is USD 60 billion due to lost productivity and healthcare needs [239]. There are differences in the seasonal and geographical occurrence of norovirus. Outbreaks occur more commonly in cold winter months, with the peaks occurring from November to April in above-equator countries, and May to September for below-equator countries [239]. Mattison et al. (2021) found that 123 rotavirus, 107 sapovirus, 10 astrovirus, and 4 adenovirus gastroenteritis outbreaks were reported in the US during 2009–2018 [240].

In the United Kingdom (UK), it is thought that around 380,000 cases of norovirus are linked to food, annually [69]. Furthermore, in Europe and European Free Trade Association (EFTA) countries, it is estimated that noroviruses cause nearly 200,000 hospitalizations every year [241]. In contrast to the US and other western countries, norovirus is rarely implicated in foodborne outbreaks in the Middle East–North Africa (MENA) region, but it is clearly present in Egypt [242]. In contrast, Kreidieh et al. (2017) reported that norovirus is an important causative agent for acute gastroenteritis among all age groups in the MENA region, but many outbreaks and cases are under-investigated and under-reported [243]. In most cases, and in order to accurately identify norovirus outbreaks, it is important to have detailed virological analyses of stool samples and epidemiological analyses of patients [244,245]. The laboratory testing of norovirus must focus on detecting its genetic material (viral RNA) or its viral antigens [246].

A large norovirus outbreak involving 176 cases linked to the consumption of raw oysters from British Columbia, Canada, was reported in 2018 [247]. Raw oysters also from the same source were recently linked to an international norovirus outbreak with 192 illnesses after oysters were distributed to restaurants and retailers in multiple states in the US and provinces in Canada [248]. Another outbreak with 60 cases of gastrointestinal illness occurred in Canada in 2022 due to the consumption of spot prawns contaminated with norovirus [233]. The Public Health Agency of Canada also investigated a large norovirus outbreak involving 339 cases associated with raw oysters from British Columbia [233]. A norovirus outbreak occurred among guests at a wedding reception in Salzburg, Austria, and it was linked to the consumption of a mushroom dish. Investigations showed that kitchen workers and guests were positive for norovirus. It was also reported that the employee and kitchen staff restroom lacked functional hand hygiene facilities [249].

Hepatitis A infection is common and frequently contributes to a more severe and prolonged epidemic, making up 2–7% of the total disease load [10]. Hepatitis A virus infection has been linked to the ingesting of contaminated raw or undercooked shellfish, ready-to-eat foods, fresh fruits and vegetables, and water [47,155,160]. A large outbreak of hepatitis A illness with over 310,000 cases including more than 8000 hospitalizations and 47 deaths happened in 1988 in Shanghai, China, and was linked to the eating of raw clams after contact with contaminated hands [250]. In 2003, another large hepatitis A illness outbreak was identified among guests of a single restaurant in Pennsylvania, US, arising from contaminated green onions. The outbreak resulted in 601 identified illnesses including three deaths and at least 124 hospitalizations [251]. In 2013, an outbreak included about 160 individuals infected with hepatitis A virus, and 69 people were hospitalized after the consumption of a frozen berry mix containing contaminated fruits from the US, Chile, Turkey and Argentina [252]. Recently, a multistate outbreak involving 19 hepatitis A virus illnesses linked to fresh, organic strawberries was reported in four states [253]. In 2023, a hepatitis A outbreak occurred with nine illnesses due to the consumption of frozen organic strawberries [254]. In Canada, an outbreak with 10 cases of hepatitis A illness was reported in 2022 due to the consumption of imported fresh organic strawberries. Another outbreak with three cases of hepatitis A was described in 2021 and was associated with frozen mangoes [233].

Hepatitis E disease is considered an important public health concern in many parts of the world [255]. The WHO estimated that there are around 20 million cases of hepatitis E viral infections annually with 44,000 deaths, and these are associated primarily with contaminated water use, most commonly in eastern and southern Asia [153]. Hepatitis E virus infections occur worldwide but are common in low- and middle-income countries, due to limited water supplies, poor environmental sanitation, and inadequate hygiene practices and health services [153]. In the UK in 2008, a hepatitis E virus outbreak occurred among passengers during a three-month world cruise, and the investigations showed that three factors including gender, age and shellfish consumption were linked with confirmed acute hepatitis E infection in four passengers. The investigations also revealed that 195 of 789 were seropositive and another 33 had elevated IgM levels, indicating that they had experienced a recent infection [256]. In China in 2018, a hepatitis E outbreak with 41 illnesses occurred due to the consumption of undercooked pig liver [257].

In the US, 123 rotavirus, 107 sapovirus, 10 astrovirus, and four adenovirus gastroenteritis outbreaks were reported to the National Outbreak Reporting System during 2009–2018 [240]. It is estimated that rotaviruses cause up to one million cases of foodborne illnesses with 15,433 cases of gastroenteritis and 34 hospitalizations in the US and are responsible for a burden of USD 18 million in direct healthcare costs and lost productivity [83]. An outbreak with 108 cases occurred among college students at a Washington DC university campus in 2000 associated with eating deli sandwiches from the university dining hall. Stool specimens were collected from students and dining hall employees, then samples were screened for bacterial, parasitic, and viral pathogens. The specimens tested were negative for any bacterial and parasitic pathogens, but were positive for group A rotavirus [258]. 

Outbreaks of sapoviruses may occur in environments that include narrowed locations, for example: nursing homes and cruise ships [100]. In 2010, an outbreak of gastroenteritis associated with sapovirus happened in Gifu, Mie, and Aichi Prefectures in Japan. The outbreak was linked to the consumption of a lunch box prepared and delivered by a catering company. A total of 655 individuals of the 3827 served developed gastrointestinal symptoms [106]. Another sapovirus outbreak with a total of 279 cases was reported in different branches of a childcare and education facility chain in Gauteng Province, South Africa in 2018, and it was suggested that the source of the virus was the catered food [259]. In one study, it was found that sapoviruses were responsible for about 4% of acute gastroenteritis outbreaks in Europe [96]. Sapovirus was detected in 8% of 2545 stool samples from acute gastroenteritis patients in Valencia, Spain between 2018 and 2020, and most sapovirus positive samples belonged to infants and children aged < 3 years [96]. Selected worldwide viral foodborne outbreaks reported in the last two decades are presented in Table 3.

**Figure 2:**
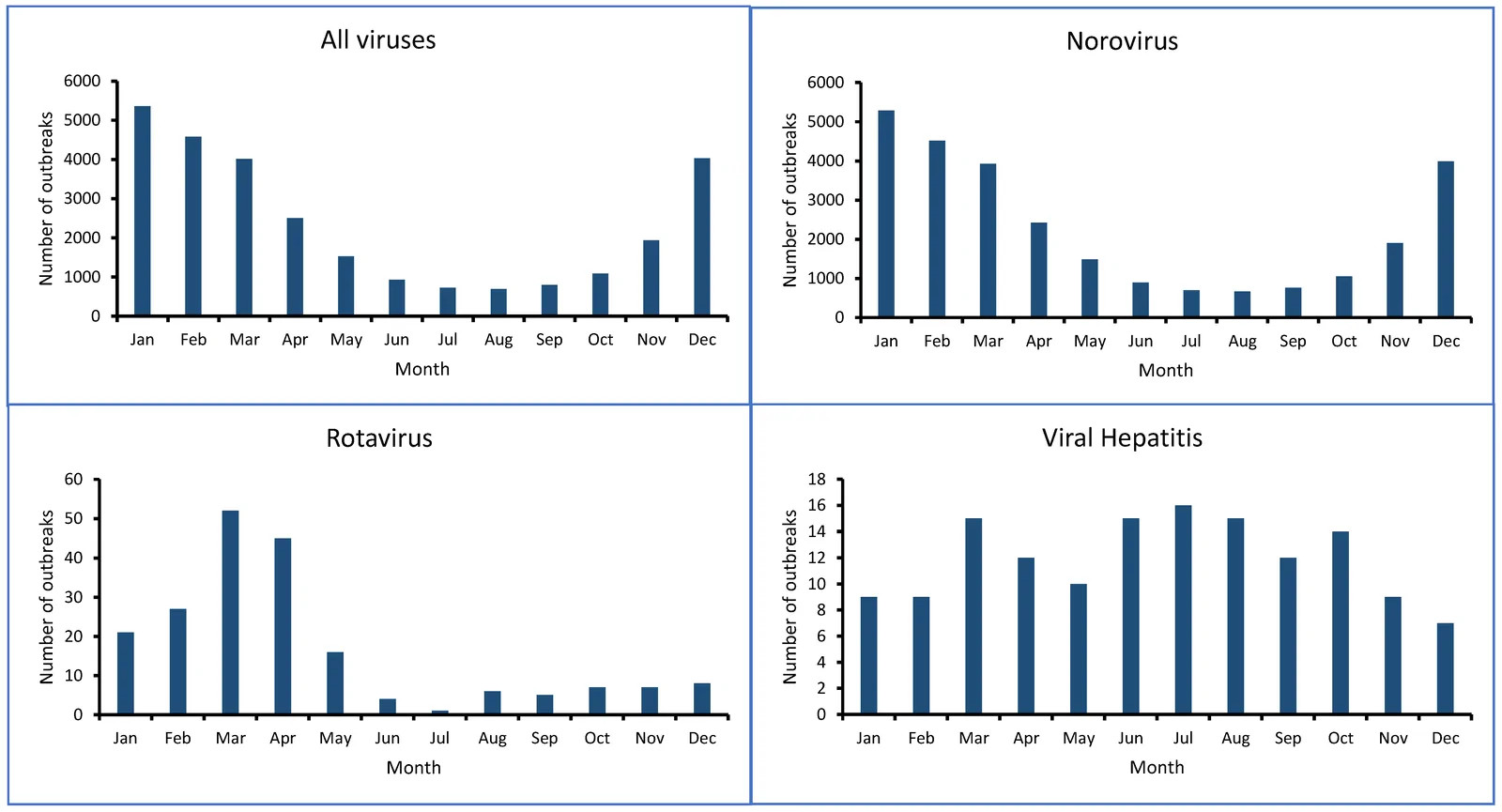
The monthly pattern of viral outbreaks reported in the US during 1970–2020 (data extracted from National Outbreak Reporting System (NORS) dashboard [228].

Seasonal variation is a recognized feature of many viral outbreaks and illnesses. However, the mechanisms underlying seasonality are still not fully understood. It appears that the persistence of viruses and host susceptibility may be enhanced at cold temperatures, and this likely contributes to the high numbers of viral outbreaks and illnesses during wintertime [281]. Sorensen et al. (2021) detected the presence of different viruses in groundwater-derived public water systems using quantitative polymerase chain reaction (qPCR) or reverse transcriptase qPCR (RT-qPCR) and found that the enteric viruses were most prevalent during November and January. Noroviruses and rotavirus are mainly responsible for viral illnesses in winter months, whereas hepatitis viruses cause illnesses during the year, with the greatest illnesses occurring in the summer semester (June to August) [282]. The monthly patterns of viral outbreaks reported in the US during 1970–2020 are represented in Figure 2.

Viruses are usually difficult to detect in food [245] because of the need to use a number of steps including virus extraction, the purification of the viral genomic material, and molecular detection. However, the detection of viruses has undergone a remarkable evolution due to reverse transcription-polymerase chain reaction (RT-PCR) technology [246,283]. RT-PCR is considered the method of choice for the virological analysis of food and water due to the small number of viruses usually present [284,285]. In addition, these low viral numbers may not be uniformly distributed, and some of the components in the food matrix may be potent inhibitors of traditional detection assays [11,33]. Typically, water, foods, or food surface samples are treated to concentrate the virus. Then, the nucleic acid is extracted and different types of PCR like monoplex RTqPCR, viability PCR, multiplex RTqPCR, digital RTdPCR and next generation gene sequencing are performed [33]. Such molecular techniques, while potentially very sensitive, do not provide information about whether the virus detected is infectious and thus capable of causing disease. The determination of infectious viruses requires other types of assays that are capable of detecting viral replication (i.e., TCID50 assays, plaque assays, focus-forming assays, cytopathology induction), all of which also could be interfered with by inhibitors in the food matrix.

## 5. The Control of Foodborne Viruses in Food Chains

Viruses present in the surrounding environment are progressively documented as a source of disease in all ages. The viruses with the most shared causes of disease being environmental contact are norovirus, hepatitis A virus, adenovirus, rotaviruses, hepatitis E virus, astrovirus, and sapovirus. Most ways these viruses become harmful to humans are via human or animal feces, sewage, or organic waste decomposition. The mismanagement of farm waste disposal can contribute where the decomposition of exposed food and crops may contaminate water sources through rain. In addition, the illegal slaughter of animals where unacceptable sanitizing or inadequate hygienic standards are used increases the risk of transmitting viruses of animal origin [46].

Most recognized outbreaks of foodborne viruses can be linked to foods that have been handled manually by a diseased food handler, rather than to foods processed industrially [228]. Emphasis should be placed on exercising good agricultural and manufacturing practices to prevent viruses from being transferred from raw materials to retail products. Bivalve shellfish should not be eaten raw or undercooked [47]. If viruses exist in food products after processing treatments, they remain contagious in most situations and in many foods for numerous weeks or days, especially if kept at or near 4 °C [125]. A variety of methods that are used against viruses and their effectiveness follow:

- Heat treatment: cooking or processing food at high temperatures can inactivate most viruses. It has been found that foodborne viruses including hepatitis A virus, norovirus, and hepatitis E virus in foods were efficiently inactivated by heat [286];
- Radiation: ionizing radiation can be used to inactivate all types of viruses in food. The US FDA approved the use of 4 kGy irradiation, which reduces viruses by about 1.0 log. Therefore, higher levels would be required to deliver control over greater quantities of viral contamination [11,16].;
- High pressure processing (HPP): the HPP treatment of foods involves treating packaged samples suspended in liquid with pressure which is rapidly released. It was found that HPP is very effective for inactivating food viruses [14];
- UV light: this technology alters the genetic material and the proteins of viruses. UV treatment is an effective method for inactivating viruses on foods or food-processing surfaces. The method is most effective in water and high aw foods [13];
- Cold plasma: cold plasma can be created by the application of an electric field to gases like helium, nitrogen, oxygen, argon, or their mixtures, which are partially or completely ionized to form reactive chemical species. Cold plasma successfully inactivated foodborne viruses including hepatitis A virus and norovirus without affecting the quality attributes of foods. This new option has significant potential value for use in the food industry [15,287];
- Pulsed Electric Field (PEF): PEF is a technique that generates a short time electrical treatment by using a pulse electric field. Although few studies have investigated the inhibitory effect of PEF against foodborne viruses, this technology may have the potential to be applied in a variety of foods [12];
- Sanitizers: sanitizers including chlorine, hydrogen peroxide and ozone showed significant efficiency in the viral decontamination of fresh produce. However, activity depended on the sanitizer type and concentration, food item, type of virus, inoculation level, and method used for decontamination [11];
- Lactic acid bacteria: Fermenting foods with lactic acid bacteria can create an acidic environment and may produce antiviral bacteriocins that could potentially be used as food additives that are hostile to viruses [288].

It is important to note that the above treatments inactivate viruses; that is, they decrease their infectivity without necessarily decreasing their genetic material. Therefore, monitoring virus inactivation by molecular techniques would not accurately measure infectivity and could lead to erroneous conclusions that the inactivation method(s) did not work. It is essential to distinguish between virus removal and virus inactivation, and this is very critical in approving the accurate steps and identifying the affecting factors that may enhance the activity of the target method to reduce the infectivity of viruses. Generally, the removal or inactivation processes of viruses should remove or inactivate the viruses’ infectivity to a greater extent than the levels of viruses in the starting materials, thus yielding safe food products [179]. With viral infections from organisms like norovirus being very common, it is prudent to prevent the contamination of the food with these viruses in the processing chain by applying strict hygienic practices. Food handlers contacting people suffering from gastroenteritis such as children are at high risk of becoming soiled and of spreading the viruses during the manufacturing of the food products. The food handlers should be conscious of practicing good personal hygiene. Increasing the consciousness of food handlers to prevent the spread of the enteric virus (often involving oral discharge) is necessary, with exceptional highlighting of the ‘‘silent’’ risk of asymptomatic ill people and virus carriers (who shed viruses after recovery) [228].

Since the potential for very large numbers of cases of viral foodborne illness occurs with each outbreak event, the adoption of strategies for the prevention and control of foodborne viral contamination is prudent [228,289]. These strategies include the following:

- Cleaning and disinfecting regular environmental surfaces touched by various individuals. The proper washing of vegetables and fruits should occur before consumption. Only potable water should contact food. Sources of water must be protected from all types of untreated wastewater contamination;
- Increasing the awareness of safety issues regarding foodborne viruses among workers at different stages of responsibility in the supply chain;
- Emphasizing good hand washing with appropriate sanitizers located near the sink. Food preparation equipment and surfaces must be disinfected regularly. Hand washing with soap and maintaining good sanitary hygiene will certainly help in reducing viral contamination [290];
- Reinforcing strict personal hygiene practices for everyone since symptomatic, colonized or asymptomatic individuals can transmit pathogens.
- Displaying clearly visible signs accompanied by frequent verbal and written reminders for food handlers to frequently wash hands after visiting the toilet and before consuming foods.
- Educating food workers and handlers about gastrointestinal illness symptoms;
- Educating the public at large about microbial safety guidelines and hygiene rules;
- Workers who are sick should not be allowed to handle the equipment involved in food processing. Employees must be made aware that at the beginning of gastrointestinal illness symptoms, it is necessity to stop working, and only re-continue work after symptoms ceased after at least 2 days;
- Retailers, distributers, and manufacturers must have an effective system in place for appropriate recalls and enhanced trace-back systems for assumed contaminated water or foods;
- Developing precise interventions for reducing the frequency of viral foodborne illness outbreaks by focusing on shellfish, produce, and food workers;
- Facilitating improvement in viral diagnostics, including the development of efficient, rapid and sensitive viral detection methods;
- Developing specific, effective viral vaccines, and antiviral sanitizers and drugs;
- Developing efficient cell culture systems and robust animal models for the recovery and identification of human foodborne viral agents.

## 6. Conclusions

Foodborne viruses constitute the leading cause of foodborne illnesses worldwide, which result in hefty public health and economic burdens. Foodborne viral infections including rotavirus, hepatitis A, norovirus, and E viruses, adenovirus, astrovirus, and sapovirus are considered major contributors to foodborne illness. with the noroviruses contributing the majority of acute gastroenteritis illnesses in humans worldwide. 

Viruses can be transmitted easily via the fecal–oral mode to contaminate water, food, and food-contact surfaces. Infected food handlers serve as a major portal for the entry of viruses into the food system. During epidemiological studies, the improper handling of food is more frequently identified as being responsible for spreading viruses than properly processed food. The incidence of norovirus outbreaks is more predominant in the cold winter season, whereas hepatitis (A and E) outbreaks are more common in summer. Hepatitis A and E viruses are quite resistant to heating, freezing, irradiation and chemical preservation approaches. Ebola virus, on the other hand, is an example of a virus that may be transmitted through the consumption of exotic meats of wild animals and by contact with infected persons. Therefore, the consumption of such meats is not recommended.

Although viruses cannot multiply in food or water, they can persist for days and even weeks in the food chain. Hence, control strategies are needed to preclude their persistence and infectivity. Strict hygienic practices such as conscientious hand washing and preventing infected individuals from coming into close proximity with prepared food, washing fruits and vegetables before preparation and consumption, plus cleaning and disinfecting surfaces are crucial interventions that limit the presence of viruses in food. Adequate food heating, vaccination against hepatitis A and E viruses and rotaviruses, avoiding the consumption of foods treated with contaminated water and avoiding eating minimally processed and exotic meats are other vital measures to prevent viral foodborne illnesses. The chlorination of water used in washing food contact surfaces, plus its use for washing fresh produce and in swimming pools, are effective approaches to control waterborne viruses. 

## Figures and Tables

**Table 1:** General and clinical characteristics of common foodborne viruses.

Discovery Date	Particle/ Genome	Genus/ Family	Structure and Size	Disease Caused	Incubation Period	Duration	Transmission	Symptoms	Prevention and Control	References
**Norovirus**										
1968	Non-enveloped/ssRNA	Norovirus/Caliciviridae	Size of 7.5–7.7 kb length and a diameter of 27 nm	Gastroenteritis	0.5–3 days	2–3 days	Person-to-person contact, fecal–oral transmission, foodborne transmission, waterborne transmission	Vomiting, watery diarrhea, abdominal cramps, fever, headache, mucus in stool, myalgia and chills	Proper hand hygiene, washing fruits and vegetables before preparing and eating, preventing infected persons from preparing food for others, cleaning and disinfecting surfaces	[10,18,33,34,35,36,37]
**Rotavirus**										
1973	Non-enveloped/segmented dsRNA	Rotavirus/Reoviridae	Large, icosahedral, and a triple-layered protein coat, up to 76.5 nm in diameter	Gastroenteritis	2 (1–4) days	3–8 (up to 22) days	Fecal–oral route	Vomiting, fever, abdominal pain, severe watery diarrhea	Routine vaccination of infants	[10,33,38,39,40]
**Sapovirus**										
1977	Non-enveloped/ssRNA	Sapovirus/Caliciviridae	Small (27–40 nm), genome of about 7.5–8.5 kb in length	Gastroenteritis	0.5–2 days	2–6 days	Fecal–oral route	Diarrhea, vomiting, nausea, abdominal cramps, chills, headache, myalgia and malaise	Cooking shellfish adequately, proper hygienic practices and sanitize surfaces with a chlorine solution	[10,41,42]
**Astrovirus**										
1975	Non-enveloped single-stranded RNA	Astrovirus/Astroviridae	Genome approximately 7 kb in size, and 38–40 nm in diameter	Gastroenteritis	3–5 days	2–3 days; recurrence possible 7–10 days later	Person-to-person contact fecal–oral route via contaminated water or food,	Nausea, diarrhea, vomiting, malaise, abdominal pain, and fever	Avoidance of shellfish from polluted waters, decontamination of food contact surfaces and good hand hygiene	[10,43,44]
**Adenovirus**										
1953	Non-enveloped double-stranded DNA with an icosahedral capsid	Mastadenovirus/Adenoviridae	Diameter of 70–100 nm, genome 28–45 kb long	Gastroenteritis, conjunctivitis	2–14 days	1–2 weeks	Respiratory or environmental routes, waterborne spread, fecal–oral route	Fever, headache, abdominal pain, vomiting, and diarrhea	Good hygiene practices, chlorinate swimming pools	[6,10,45,46]
**Hepatitis A**										
1973	Non-enveloped or quasi-enveloped/ssRNA	Hepatovirus/Picornaviridae	Size of 7.5 kb and a diameter of 27 nm	Hepatitis	2–4 weeks (15–50 days)	2 months	Fecal–oral route	Nausea, anorexia, diarrhea, vomiting, malaise, and myalgia. Other symptoms may be present, such as: light-colored stools, dark-colored urine, jaundice	Vaccination, good personal hygiene, avoidance of eating raw shellfish, prevent infected persons from preparing food for others, cooking foods and heating drinks for at least 1 min at 85 °C (185 °F) inactivates hepatitis A virus	[10,33,47,48,49,50]
**Hepatitis E**										
1983	Non-enveloped or quasi-enveloped/ssRNA	Orthohepevirus/Hepeviridae	Diameter of 27–34 nm, size of ∼7.2 kb in length	Hepatitis	2 weeks to 2 months	4 weeks (2–18 weeks)	Fecal–oral from water and food	Jaundice, vomiting, diarrhea, and abdominal pain	Good sanitation, vaccination which is only available in China	[10,33,51,52,53,54,55,56,57]

**Table 2:** Numbers of outbreaks, illnesses, hospitalizations and deaths associated with viruses spread by water, food, person-to-person contact, environmental sources, animal contact and unknown sources in the US from 1971–2022.

Foodborne Virus	Contamination Route	Outbreaks	Illnesses	Hospitalizations	Deaths
**Norovirus**	Foodborne	6662	164,740	1664	17
	Waterborne	130	21,341	77	1
	Person-to-person	18,996	663,775	9652	887
	Environmental contact	58	2679	20	1
	Animal contact	0	0	0	0
	Unknown	1775	46,162	753	51
**Viral Hepatitis**	Foodborne	109	3051	479	11
	Waterborne	34	894	17	0
	Person-to-person	0	0	0	0
	Environmental contact	0	0	0	0
	Animal contact	0	0	0	0
	Unknown	0	0	0	0
**Rotavirus**	Foodborne	17	449	7	7
	Waterborne	1	1761	0	0
	Person-to-person	156	3515	125	11
	Environmental contact	0	0	0	0
	Animal contact				
	Unknown	25	512	10	2
**Adenovirus**	Foodborne	2	11	0	0
	Waterborne	4	708	1	0
	Person-to-person	16	350	29	3
	Environmental contact	0	0	0	0
	Animal contact	0	0	0	0
	Unknown	3	36	0	0
**Astrovirus**	Foodborne	3	49	1	0
	Waterborne	0	0	0	0
	Person-to-person	25	1505	7	0
	Environmental contact	0	0	0	0
	Animal contact	0	0	0	0
	Unknown	5	80	0	0
**Sapovirus**	Foodborne	20	294	3	0
	Waterborne	0	0	0	0
	Person-to-person	157	6926	26	2
	Environmental contact	0	0	0	0
	Animal contact	0	0	0	0
	Unknown	22	952	7	1
**Other viruses**	Foodborne	103	3049	24	0
	Waterborne	1	36	0	0
	Person-to-person	0	0	0	0
	Environmental contact	0	0	0	0
	Animal contact	0	0	0	0
	Unknown	0	0	0	0
**Unknown viruses**	Foodborne	0	0	0	0
	Waterborne	3	7	0	0
	Person-to-person	0	0	0	0
	Environmental contact	0	0	0	0
	Animal contact	0	0	0	0
	Unknown	0	0	0	0

Data extracted from National Outbreak Reporting System (NORS) Dashboard [231]. NORS includes data starting in 1971 for waterborne outbreaks, 1998 for foodborne outbreaks, and 2009 for other types of outbreaks.

**Table 3:** Selected worldwide norovirus and hepatitis A virus foodborne outbreaks in the period 2002–2022.

Virus	Year	Country	Food item	Illnesses	Hospitalizations	Deaths	Reference
**Hepatitis A virus**	2002	New Zealand	Raw blueberries	81	18	1	[260]
	2003	USA	Green onions	601	124	3	[251]
	2009	Australia	Semi-dried tomatoes	562	253	1	[261]
	2010	France	Semi-dried tomatoes	59	28	0	[262]
	2010	Netherlands	Semi-dried tomatoes	13	0	0	[263]
	2012	Canada	Frozen pomegranate arils	9	0	0	[264]
	2012	Germany	Bakery products	83	ND	ND	[265]
	2013	10 European countries	Frozen blackberries and redcurrants	1444	ND	0	[266]
	2013	USA	Pomegranate seeds	165	71	0	[267]
	2016	USA	Frozen strawberries	143	56	0	[268]
	2016	USA	Raw scallops	292	74	0	[269]
	2018	Australia	Frozen pomegranate arils	30	25	1	[270]
	2018	Australia, Sweden	Frozen berries	34	ND	ND	[271]
	2020	China	Shellfish	110	ND	ND	[272]
	2021	Canada	Frozen mangoes	3	2	0	[273]
	2022	New Zealand	Raw blueberries	32	14	0	[274]
	2022	USA	Fresh organic strawberries	19	13	0	[253]
	2022	Canada	Fresh organic strawberries	9		0	[233]
	2023	USA	Frozen organic strawberries	9	3	0	[254]
**Norovirus**	2002	Italy	Raw mussels	103	ND	ND	[275]
	2005	Denmark	Frozen raspberries	400	23	0	[276]
	2006	Sweden	Frozen raspberries	12	ND	ND	[277]
	2009	Finland	Frozen raspberries	46	ND	ND	[278]
	2010	Denmark	Lettuce	264	ND	ND	[279]
	2012	Germany	Frozen strawberries	11,000	38	ND	[280]
	2016	USA	Unknown	45	0	0	[231]
	2018	USA	Oysters	100	1	0	[231]
	2018	USA	Raw oysters	16	2	0	[231]
	2018	Canada	Raw oysters	176	ND	0	[247]
	2022	USA	Raw oysters	192	ND	0	[248]
	2022	Canada	Spot prawns	60	ND	0	[233]
	2022	Canada	Raw oysters	339	ND	0	[233]

ND: not determined.

## Data Availability

Data sharing not applicable to this article as no datasets were generated or analyzed during the current study.
